# Supplementary material for: Genetic Analysis of SCN11A, SCN10A, and SCN9A in Familial Episodic Pain Syndrome (FEPS) in Japan and Proposal of Clinical Diagnostic Criteria
Source: Int J Mol Sci. 2024 Jun 21;25(13):6832. doi: 10.3390/ijms25136832 (PMC11241565; doi:10.3390/ijms25136832)
Supplement: Supplementary file 1 [file ijms-25-06832-s001.zip › ijms-3013332-supplementary.pdf]

Supplementary

Table S1. Primers used for the sequence analysis

Primers for *SCN9A*

exon	Forward PCR Primer (5' > 3')	Reverse PCR Primer (5' > 3')	Size	Sequence Primer
2	GCCCATTGCCTGACACATAG	TTATACAGAAGGAAGCCAACAGA	640	Forward
3	CATTGCTGGAGTCTGATGGC	CACCATAAAGTGCCACTGGA	362	Forward
4	AGCATTTCAACAAGTGTCATCCT	CTGGCAGGAAAAGGAAAGGA	292	Forward
5	CCCAACAGCCATCCTCCAAT	GAGGTTTGCTGTTATTGGAACA	400	Forward
6	GCCCCAAACGTAGAAAATACCT	GACGACACACACACAAACGA	327	Forward
7	TGCATGACTTTCTAGGAAAGCT	GACCCAAATTCACACTGTAGCA	540	Forward
8	TGGGACCAGGCCTGAATTT	AGACTGGATTTGCTGTGGGA	332	Forward
9	CCCCTATAGAAGAAACCTTGAGT	AGGCTCTTAACATACACCAGGT	380	Forward
10	GGACTATGGATATCACCTGTTCC	AAGCATATACCGCAGAGCCT	766	Forward
11	TGCTAATAGGTGAAAGATGTCCT	TCTCCCGAGTTCTCTTACCC	559	Forward
12	GTGCAGCAAAATGAATGGCA	GCAGAGCCATTCACAAGACC	953	TATCAGTAGGTGCTTCAG
13	GCAGAGAGGTGATGATAGTGATGAT	GTCTATTTCATGAGTCCTCCATAAA	513	ATATAGTGACAGAATCAC
14	TGCTTCATCTAGGCAACGAA	GCAGCAATGTAATTAGGAAGGTG	624	Forward
15	AGCCAGGGAAGAGCAATACCTAA	TTGAGCATGTCTTCAAAAATTG	556	Forward
16	AGGACTTAAAGTCTGCTTTACCC	CCTCTGCCACTGTAATGTAAAAG	1112	Forward
17	TGGCCTATATTCGGAAAAGTG	GTCACGCACAAAAACTATCCA	699	Forward
18	ATTGCAAATAGGGTATAGGTTGTC	AGGACCAGAGTACAGACTCTTTCA	846	Forward
19	CTGAAATTGCAAGTATAGGTGTTG	TGGCATGTAGGAGTGCTCAGTAA	465	Forward
20	AGCAGCAGCACTGGTGAGAA	TCTGCCCTTTTATCATCTCCT	624	Forward
21	GTTCCTGTTGAGTTGCTTTTAGTG	CTGCTAATTGAGTATCTTTTCAGGC	1270	Forward
22	AGTCGGCTGGTTGGTTTGA	ACCTCATGATCTGCCCACCTT	591	Forward
23	GGACATGTTGAATACAGCAAAAC	GTTGTAGAGCTTGGATGATATGG	400	Forward
24	CACTTAGTTATTGAATTTTCCTACCA	CTGTGCAAAAATGAATAAGATTAAGC	625	Forward
25	TGGGAGTTCACTCATGATTTGG	CCATGTTGAGACAGATAAGAACCA	763	Reverse
26	AAAAGCCAATTCCTCGACCA	CCCAGAAACACTGTAGTATGAGAGA	611	Forward
27	CTTGAATTCATAAGAAATGAGTTGAC	CCCAAACCTCATAGAACATCTCA	692	Forward
	GAAGGAGACTGTGGTAACCCA	CTGTGAAAAGATGACAAGGCA	1119	Forward

Primers for *SCN10A*

exon	Forward PCR Primer (5' > 3')	Reverse PCR Primer (5' > 3')	Size	Sequence Primer
1	AAGGGCTGTTCTGACAATCAA	TTTAGCAGACTGCCACATCAC	506	Forward
2	CCTTCTTGCTCATAAGCCTGA	TTGGATAAGGGCTCTGTTGCT	326	Reverse
3	AATCATTCAGCATCAAGGTGA	AGCATTACCCACATGAGGCAT	273	Forward
4	TGGAAGAAGGAACCCCCTGA	TCTTGGAGCAAACGTTCATG	330	Reverse
5	TGTCCATTCTGCATCCTTTC	ACAATAGTCTTTGCCCTGGAA	306	Forward
6	TGAAGGAGGATGGCTAAGATG	TCCATATCCCCTGTCCCTATA	350	Forward
7	TGGAAGACTTGGGAATATTGC	ATCTTTCCCAGGACCTGCAA	264	Reverse
8, 9	AAACTCTGTGGTTGTCTCGCA	AAACGGTGCCCTAATTGAAG	658	Forward, Reverse
10	TGTTTCAGATCTCTCACCCCT	TGTCTGGATCCTTTTAGGCTC	400	Forward
11	ATAAAGACAGGGGCAGGGAAA	AAGATCCCTTCCATTGGTGA	494	Forward
12	GCCAATAAGAGGCTGAATGAT	TCCAGTACACAGAGATGGACG	306	Forward
13	GGGCTGAAGTCAAAATGTTGG	TGCTGCTCTGCTCTCAGCTAA	501	Forward
14	GACATTTTCTTGCTGCCATTT	CCACAAGGACAGGAGAGGAA	364	Forward
15	TGAGAGGCCTTGTGTTTTTCC	CTGGGCATAAAAATGCAGATC	561	Reverse
16	TTGCCAGCCAGCTGCTAA	AATGGGCAGGACCTGCTGAA	622	Reverse
17	TGATGTGCAAGATCCCTTCA	TGTGATTGAGTGCAGTCTGGA	338	Forward
18	TGGCGAGGCACCAAACAT	AAGTGGGCACAGCTTGTTTGT	338	Forward
19	TTTTCCAAGGGCATGACAAG	TTGGGGACTTTCAGCTCAGTA	386	Forward
20	TGACTACCAAATCCCCTTCCA	TTCTTTGGAGTAGCCCTTGAG	407	Forward
21	TGGCTTAGACGTGTAGGAATG	AGGGTGGTTTTGAACTCCTAA	354	Forward
22	CTCATGAAGCAGCCTGAATGA	TCCTTGCAAAGTCCCCACATA	481	Forward
23	AGCCCTTTCCAACACAGGACT	AGTGTGAGGTTGCTGGGTGAT	292	Forward
24	AGATAAATCACCTGCACCCAG	TGATGGGCTGTGAGTAGTGTT	355	Forward
25	AGGAGGGGAGAAGGTTGATAT	TCCATAAACAGCACTCCCTCA	309	Forward
26	TGGAGTGTGATGGAGACAAAA	GTTGGTTGGTTATTTCCTTGG	458	Forward
27	CAAATGTGGATGCCCAAGTG	TGTGACCAGTGGCATGCATT	1450	Forward, Reverse<br>TACTGTGACCCCAATCTGCC

Primers for *SCN11A*

exon	Forward PCR Primer (5' > 3')	Reverse PCR Primer (5' > 3')	Size	Sequence Primer
2	AACTCAACTTCCCGCCTTTT	CCTGCCTGCTATAAACCCTAA	649	Forward
3	TTCCACAGCCCAACAGTTTAT	ATCTTGCTCACATCCAGCACA	410	Forward
4	TAGATGCACCTGCAGAAAAGG	GGACAGGTGAGTGAAGGAAAA	239	Forward
5	GGAAAAAAAAATCAAGGCCAG	TTCAGGGCCAACCAACACA	371	Forward
6*	ACAGTGGTATTGCCAGATCCT	TCAAGCAGTTAGCACAGTGCC	451	Forward
7	AGCACAAACTCAAGGCATCA	GAAGCCAAAGAATGAGGCAA	519	Forward
8	TCTTGGGCTCCAGTTTCTCTC	TGCCTTAAACACCAGGAAATG	406	Forward
9	ACAGTGCAGTGGGCCACTTTA	AAAAGTGGGGGATGAATGGT	411	Forward
10	GGGAAGCTATATGGTCTGTGG	TGAAAGATAGCAGCACAGCCA	519	Forward
11	CAGAGCTAGCGTAGTCCAGGT	AGACACATGGATGCATGAAAG	471	Forward
12	TAAGTTTTGAGGAGAGGCAGC	TTCCAAAACAGCCTCCTTTG	296	Forward
13	TGGTGGAAAAACCTTTCTGAC	AACAGCCATCTTTTCCCTCA	539	Forward
14	TACTTCCCTTGGGCCATTCTT	TCAGTTAAGCGAAGTCCCCC	446	Forward
15	GCGATCTGATGGCTATGTTCA	AATTTGGGGGCATCAGTCAA	755	Forward
16	GCTTTCTTAGGAGACAGTGGG	CAGCCACGTTTTGTACCCTTA	688	Reverse
17	TGTTGAGTTTTTCCAGGAGCA	AAAGTTGAGAAACCTGGCCT	426	Forward
18	TCATCCTAGAAACCTTTGCCT	CGACAATCGCCTCAAGAATCT	397	Forward
19	ACATGGCAGGGACCAGATTTA	AACCAGCAGCATCAGAGACCA	453	Reverse
20	TTGAGCAGAACCTCATTACGA	AGACATCCATATGCGGCACA	403	Forward
21	GAGTGCATTGTTCCCACGTTT	CAAATTCTAAGCACTGGGCA	356	Reverse
22	ATGGGGCACATGTATGTGGA	TTCCAGAAGGCATTGGCTTT	550	Forward
23	TCCTAGAAGGTTGGGGTATCA	AAACAAGGCAGAGAACCCCAG	298	Forward
24	TGGCTGTTAGGAAGAGGCTAT	TTTGAGGATCATCTCCAGGCA	431	Reverse
25	TTCGCTATGGTCCAGTCTCTT	GAAGGATGGCATTACGGAAA	337	Reverse
26	AGCCCAAGCACTTAAAACCA	AGAATGGCATTAGTACCCCTG	649	Reverse
	ATTGATAAAAAGTTGGCCCC	CTGAGCAGGGAATCCCAA	450	Forward
27	CAGAGTCTGGAATCGATGACA	ACGCAAAGGCTCAGGCAA	404	Forward
	AAGTGTGGGAAAAGTTTGACC	TACACTAAGCTGCTGACCCCT	574	Forward
